# Imported malaria in an area in southern Madrid, 2005-2008

**DOI:** 10.1186/1475-2875-9-290

**Published:** 2010-10-20

**Authors:** Sonia Rey, Inés Zuza, Belén Martínez-Mondéjar, José M Rubio, Francisco J Merino

**Affiliations:** 1 Microbiology and Parasitology Department. Severo Ochoa University Hospital, Avda Orellana s/n, 28911 Leganés, Madrid, Spain; 2 Preventive Medicine Department. Severo Ochoa University Hospital, Avda Orellana s/n, 28911 Leganés, Madrid, Spain; 3 Malaria & Emerging Parasitic Diseases Laboratory, Parasitology Department, National Centre of Microbiology. Instituto de Salud Carlos III, Cra. Majadahonda Pozuelo Km.2, Majadahonda, 28220 Madrid, Spain

## Abstract

**Background:**

In Spain, malaria cases are mostly due to migrants and travellers returning from endemic areas. The objective of this work was to describe the malaria cases diagnosed at the Severo Ochoa University Hospital (HUSO) in Leganés in the south of the Madrid Region from 2005 to 2008.

**Methods:**

Descriptive retrospective study performed at HUSO. Data sources are registries from the Microbiology Department and malaria cases notified to the Preventive Medicine Department. Analysed parameters were: administrative, demographical, related to the stay at the endemic country, clinical, microbiological diagnosis method, pregnancy, treatment and prophylaxis, co-infections, and days of hospital stay.

**Results:**

Fifty-seven patients diagnosed with malaria were studied. Case distribution per year was 13 in 2005, 15 in 2006, 15 in 2007 and 14 in 2008. Thirty-three patients were female (57.9%) and 24 male (42.1%). Mean age was 27.8 years. Most of the malaria cases were acquired in Nigeria (49.1%) and Equatorial Guinea (32.7%). 29.1% of the patients were immigrants who had arrived recently, and 61.8% acquired malaria when travelling to their countries of origin to visit friends and relatives (VFR). Majority of cases were diagnosed between June and September. Microscopy was positive in 39 cases (68.4%) immunochromatography in 42 (73.7%) and PCR in the 55 cases where performed. *Plasmodium falciparum *was responsible for 94.7% of the cases. The more frequent symptoms were fever (77.2%), followed by headache and gastrointestinal symptoms (33.3%). Nine cases needed hospital admittance, a pregnant woman, three children, four VFR and an African tourist, but all evolved favourably. Chemoprophylaxis data was known from 55 patients. It was taken correctly in one case (1.8%), in five (9.1%) the prophylaxis was improper while the others 49 (89.1%) cases had not followed any anti-malarial prophylaxis.

**Conclusions:**

Children, pregnant women and the VFR have the highest risk to present severe malaria and to need hospital admittance. Another important risk factor for acquiring malaria is incorrect prophylaxis. The first place for malaria acquisition was Nigeria and the main species causing malaria was *P. falciparum.*

## Background

Approximately, 50% of the world's population, mostly that living in the world's poorest countries, is at risk of malaria. In 2006, there were an estimated 247 million malaria cases and an estimated 880,000 deaths from malaria. Most cases and deaths are in sub-Saharan Africa, mostly in children under five years of age. However, Asia, Latin America, the Middle East and parts of Europe are also affected [1]. Travellers from malaria-free regions going to areas where there is malaria transmission are highly vulnerable. They have little or no immunity and often they are exposed to delayed or wrong malaria diagnosis when returning to their home country [2].

In 2007, international tourist arrivals grew to a new historical maximum of 900 million [3]. In agreement with the statistics for Spanish Tourist Movements (FAMILITUR), during 2007, 23.2% of the foreign destinations were outside Europe, meaning 2.6 million people [4]. Also, more than two million non-UE live in the country, being susceptible to suffer from tropical diseases [5].

The last case transmitted by vectors in Spain was diagnosed in 1961, but no cases of vector transmission have been reported since [6]. All described cases have been imported (tourism, immigration coming from endemic areas, international cooperation projects) [7], except airport malaria cases, congenital transmission and induced malaria cases (by injection or blood transfusion from infected patients, or by the use of contaminated needles and syringes) [8,9].

In Spain, 345, 342 and 338 cases were reported in 2007, 2008 and 2009 respectively. In 2007 the incidence rate (IR) was 0.8 cases/100,000 people, while in the Community of Madrid with 114 cases, IR was 1.9 cases/100,000 people, 2.4 times higher than the Spanish IR. Health areas in the Madrid Region with higher rates of incidence were those corresponding to Leganés- Fuenlabrada (southern area), and Alcalá de Henares-Torrejón de Ardoz (north-eastern area), with 8.0 and 4.0 cases per 100,000 people, respectively [10].

The objective of the study is to describe the malaria cases diagnosed in the Severo Ochoa University Hospital (HUSO) placed in Leganés in the south of Madrid Region during the 2005-2008 period.

## Methods

### Population and period of study

Studied population was all patients with parasitological diagnosis for malaria between January 2005 and December 2008. Patients come from the area covered by HUSO, which included Primary Care Units of Leganés and Fuenlabrada. On 1st January 2008, Leganés population was 188,676 people, out of which 23,858 were immigrants, representing 12.7% of the total registered population [11]. Fuenlabrada population, in the same date, was 194,791 people out of which 23,881, representing 12.3%, were no native population [12].

### Epidemiological survey

The study was performed as a descriptive retrospective survey performed in the HUSO. Data sources were records from the Department of Microbiology and malaria cases notified to the Preventive Medicine Department. A database was designed, completed after the retrospective review of the Microbiology registries, discharge report (for patients with admittance) and notification sheets from the Preventive Medicine Department.

The main parameters analysed were as follows: administrative (number of clinical story, date for admittance and discharge, and admittance service), demographical (date of birth, sex, nationality, country of origin and residence), data related to the stay at the endemic country (country, duration, date of departure and date of entry in Spain), clinical (symptoms and clinical signs of presentation, complications), laboratory data (haemoglobin, haematocrit, platelets), parasitological diagnosis method (microscopy, antigen detection and PCR, including *Plasmodium *species and parasitaemia -<250 very low trophozoites/μl, 250 to 20,000 low, 20,000 to 50,000 moderate and >50,000 high-), pregnancy, treatment, co-infections (parasites, serology), intake or not of prophylaxis and days of hospital stay in case of admittance.

The diagnosed cases were divided in three groups: 1) immigrants recently arrived to Spain (less than six months of stay), 2) people having resided in Spain for more than six months, travelling to endemic areas to visit relatives and friends (VFR, be the traveller Spanish or not) and 3) people travelling to an endemic area for tourism, business, cooperation or others.

### Laboratory

Parasitological diagnosis was performed by microscopy and antigen detection (Binax NOW ICT) in the 57 cases analysed and by PCR in 55 of the cases [13] (performed on the Malaria & Emerging Parasitic Diseases Laboratory, National Centre of Microbiology at Majadahonda from the Spanish Ministry of Science and Innovation).

### Statistical analysis

A descriptive analysis was performed, presenting the percentages to describe the qualitative values, as well as means with their standard deviation (SD) for quantitative values. Data were processed using SPSS 15 software.

## Results

### Group characteristics

Fifty-seven patients diagnosed with malaria were studied. In two patients, diagnosis was performed at the Hospital Carlos III in Madrid, with data submitted to the HUSO because they originated from this health area. Serological and haematology data for these patients were unknown. Also, for another two patients the only known data was birth date, sex, diagnosis method used and *Plasmodium *species.

Case distribution per year was 13 in 2005, 15 in 2006, 15 in 2007 and 14 in 2008. Twenty-four out of 57 (42.1%) were male, while 33 patients were female (57.9%), of which four were pregnant. The mean age was 27.8 years, with 11 patients under 18. Six out of 55 patients (10.9%), of whom nationality was known, were Spanish. The other 49 patients (89.1%) came from sub-Saharan Africa countries; 22 (40.0%) from Nigeria, 19 (34.5%) from Equatorial Guinea, 6 (10.9%) from Cameroon, one (1.8%) from Ivory Coast and one (1.8%) from Mali. Out of the six patients born in Spain, one was an adult tourist returning from India; three were brothers whose parents were Nigerian and the other two were the spouse of people coming from Ivory Coast and Nigeria. Out of the 49 foreign patients, 19 (38.8%) were recently arrived immigrants with residence in Spain for less than 6 months, one was resident in Equatorial Guinea (2.0%), who came as a tourist to Spain, and 29 (59.2%) had a longer resident period, between six months to 20 years. In one case, the residence time was unknown.

**Figure 1:**
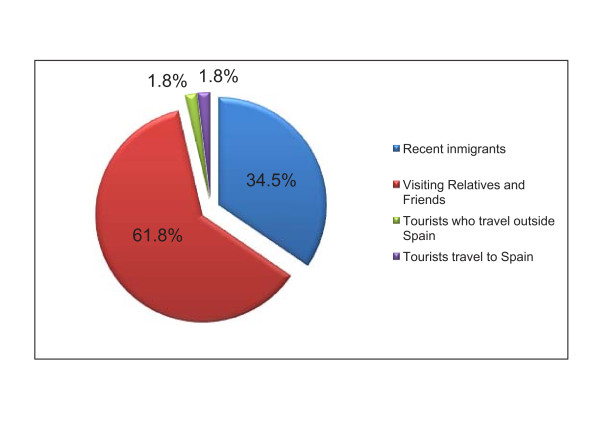
**Classification of patients by type of traveller**.

The more numerous group of patients was the VRF (61.8%), where both the immigrants with long residence in Spain (29 cases: 52.7%) plus their spouses and children born in Spain (5 cases: 9.1%) are included, followed by the immigrants recently arrived from endemic areas (19 cases: 34.5%), and finally, a minority group for tourists (Figure 1).

Regarding the duration of travelling of the VFR and the Spanish tourist, out of the 35 patients staying in any endemic country, three people remained one year, for three people there are not data and for the remaining 29, mean duration was 31 days (SD: 19.0).

**Figure 2:**
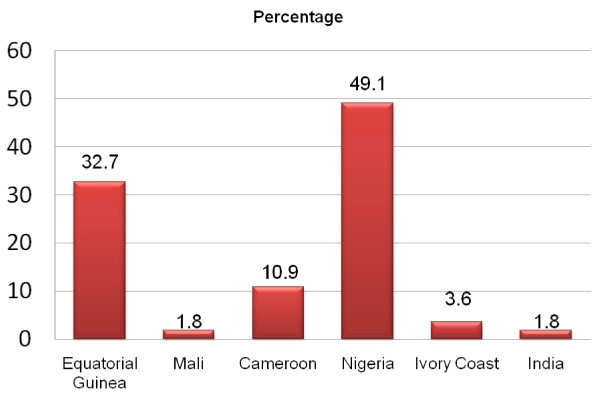
**Endemic countries where malaria was acquired**.

The acquisition of malaria for the 55 cases for whom the country was known was mainly Nigeria (27 cases: 49.1%) and Equatorial Guinea (18 cases: 32.7%). The rest of cases were acquired in Cameroon (6 cases: 10.9%), in Ivory Coast (2 cases: 3.6%), in Mali (1 case: 1.8%) and in India (1 case: 1.8%). The patients acquired malaria in their own country or in the family's native country. Only one Spanish adult tourist and a sub-Saharan African tourist became infected in an unrelated country (Figure 2).

**Figure 3:**
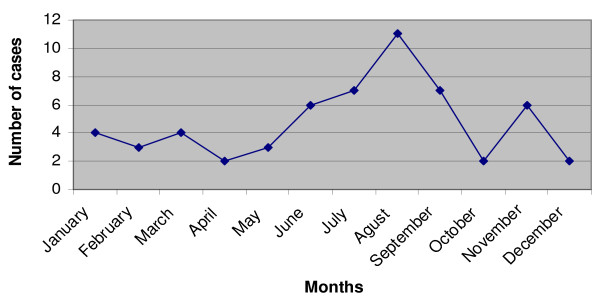
**Distribution of months where malaria parasitological diagnosis was performed**.

Most of the cases were diagnosed between June and September coinciding with the summer holydays period. There is a progressive increase in the number of positive diagnosis cases during summer along June to August and starting to decrease in September (Figure 3).

### Laboratory

The diagnosis by microscopy was positive in 39 out of 57 cases performed (68.4%), the immunochromatography test in 42 out of 57 cases (73.7%), and the PCR in the 55 cases (100%) where it was performed. In three patients, malaria diagnosis was performed more than one year after the date of return from the endemic country and in one case five months later, none of these were from Spanish origin. For these cases, PCR was the only test rendering a positive result.

The mean for the time lapse between the onset of symptoms and parasitological diagnosis was 4.5 days, while the lapse between the date of return from the trip and the diagnosis was 21.5 days. A special case was a patient with *Plasmodium ovale *infection who was diagnosed 277 days after travel return. The mean for the time lapse between the return date and the diagnosis for patients without symptoms was 112 days, with a maximum of 468 days.

*Plasmodium falciparum *was characterized in 54 cases (94.7%), while *P. ovale *was present in the other three cases. The mean parasitaemia was 32,245 trophozoites/μl of blood (mean 12,500 and typical deviation 45,670). Parasitaemia was very low in 12.5% of the cases, low in 46.9%, moderate in 15.6% and high in 25.0%.

Six patients showed single or multiple co-infection with other parasites (*Mansonella perstans *(5), *Giardia lamblia *(1), *Ascaris lumbricoides *(2), *Trichuris trichura *(2), *Onchocerca volvulus *(1)). Furthermore, three patients were co-infected with chronic viral diseases, one out of the 20 patients analysed was infected with HIV, one with chronic HBV and one with HIV plus HCV.

### Clinical data and treatment

Forty-six of the cases presented malaria symptoms (80.7%), nine patients did not show any symptom (15.8%) and for the two remaining cases this data was unknown. Most frequent symptoms were fever (77.2%), headache and gastrointestinal symptoms (33.3%), followed by asthenia (15.8%). Other described symptoms included arthralgia, malaise, hepatomegaly, jaundice and anaemia.

The haematological data was known in fifty-one patients. Haemoglobin (Hb) ranged between 6.9 and 16.1 g/dl, 29 patients (56.9%) had a Hb value lower than 12, while just one had a Hb lower than 7. Haematocrit (Hto) ranged between 22.1 and 45.2%, presenting a Hto lower than 37% in 34 patients (66.7%). Platelets ranged between 37,000 and 584,000 with a mean of 186,392, with 25 patients (49.0%) presenting thrombocytopaenia <150,000 platelets/μl.

Nine patients needed hospital admittance, a pregnant woman, three children (3, 12 and 15 years old), four VFR and the African tourist, with a mean stay of 7.5 days (SD: 4.5). None of the patients presented any complications.

Treatment data was available for 14 patients, with quinine and doxycycline (57.1%) being the most frequently used, followed by quinine and clindamycin (28.6%), atovaquone/proguanil (7.1%) and chloroquine (7.1%). Treatment was completed with primaquine in the three cases with *P. ovale *infection. Chemoprophylaxis data was known from 55 patients. It was taken correctly in one case (1.81%), whereas in five cases (9.09%) the prophylaxis was inadequate. The remaining 49 (89.1%) cases had not taken any anti-malarial prophylaxis.

## Discussion

Malaria was eradicated in Spain in the early 60 s of last century. Since then, very low or null incidence has been reported until recently years, where due to increase of tourism and immigrants from malaria endemic countries, the number of reported cases has increased to near 400 per year [7].

This study described the epidemiology of malaria in imported cases, diagnosed in the Reference Hospital of a health area in the south of Madrid, where about 15% of the population is immigrant, including a high number of natives from endemic malaria countries. This population pattern confers a special characteristic to this area, which differs from other Spanish health areas.

All patients, except a Spanish tourist infected in India, acquired the infection in a sub-Saharan Africa country, in agreement with the fact that this continent is where malaria acquisition is more frequent [14-16]. Except for the Spanish tourist, all patients were immigrants or relatives, such as their children or their spouses. The southern Madrid region is a middle- and working-class area, where very few Spanish residents have the capacity to travel to tropical countries. On the other side, the immigrant population go for the summer holidays to their native countries, where they become infected and are diagnosed upon return, which fits with the observation that most of the malaria diagnosis is performed during the summer period [17-19], and also with the fact that in this series there was a progressive increase in the number of positive diagnosis cases between June to August.

For nearly 95% of the patients in this study, the species causing malaria was *P. falciparum*, which shows a higher prevalence than that observed in France (80%) or in Italy (73.3%), where most cases are also imported from sub-Saharan Africa countries [18]. Instead, other countries as UK or Australia, have a higher number of immigrants coming from South-Asia, have *Plasmodium vivax *is the prevalent species, present in over 50% of the cases diagnosed [18,20].

It is often reported in Spain that most of malaria imported cases were acquired in Equatorial Guinea, a small country in West Central Africa with 600,000 inhabitants and a former Spanish colony. Reports carried out in Barcelona and Valencia, two of the biggest Spanish cities, show that Equatorial Guinea was the place were more cases are acquired with 35.9% and 40% of the total cases respectively [15,20]. Moreover, in a long retrospective study performed in Barcelona, 57% of infected African immigrant came from this country [16]. Infected immigrants from this country represent 32.7% of the cases in the series presented in this work, which is similar to previous report, but with the difference that the first place of acquisition was Nigeria (49.1%). For Leganés and Fuenlabrada, these data correlated with the inhabitants of every nationality in the local census (1,357 from Equatorial Guinea and 3,728 from Nigeria in 2008), but not in Barcelona where there were only 1,889 inhabitants from Equatorial Guinea and 3,453 from Nigeria in the same year [12]. The census data show that the immigrants tend to stay in districts or towns by nationalities. For example, in Leganés, 46% of sub-Saharan Africa immigrants come from Nigeria, while only 17% are from Equatorial Guinea. In Madrid, the biggest Spanish city, only 13% come from Nigeria, while 40% come from Equatorial Guinea. These variations into the population must be taken in consideration to analyse the pattern of malaria in immigrants in each health area.

Malaria is an infectious disease affecting health in a variable way. Residents in endemic areas acquire a certain degree of immunity after repeated exposure to parasite where frequency and seriousness of malarial episodes decreases with age [14]. It is thought that immunity diminishes once the exposure to the parasite ceases and this has its reflection in the date presented here, where the 61,8% of infected patients were VFR, which is a similar percentages to previous reports [16].

Several patients (15.8%) were diagnosed without presenting any symptom. These asymptomatic patients can be an important factor in a possible autochthonous transmission of the disease. Most of the Mediterranean countries, where malaria has been eradicated, are still susceptible to its reintroduction due to the persistence of the vector [7] and several cases of autochthonous transmission has been reported in Greece, Italy and France in the last years [21-23]. These asymptomatic patients, probably due to the development of partial immunity, keep the parasitaemia to a very low level making the diagnosis by traditional methods difficult [24]. The implementation of PCR-based methods for malaria diagnosis, capable to detect cases with a very low of parasitaemia, allows a more accurate diagnosis in people with some degree of immunity [13]. This is especially important for pregnant women coming from endemic areas to avoid mother-child transmission and to stop horizontal transmission by blood donor or organ transplantation [8,25].

The asymptomatic patients suffer long delays in the diagnosis with an average of 112.7 days after travel return, while for the symptomatic patients the delay was 21.5 days. This value for correct diagnosis is still too high, but it was due to a *P. ovale *infection, which was diagnosed 277 days after return. Removing this case results in an average between diagnosis and travel return of 14 days. The delay in the correct diagnosis of the diseases is directly related with the severity of the symptoms and the correct evolution of the patients.

The risk of complicated malaria is higher in children and pregnant women in endemic areas and in travellers coming from a non-endemic area, who do not have a previous immunity against the parasite [14]. People in this study included in the risk category were nine children, four pregnant women and three Spanish adults. Out of them, only a pregnant woman and three children required admittance to hospital. The three children were immigrant newcomers to Spain and the woman was living in Spain and returned to visit relatives in Nigeria. Another five people were admitted in the Hospital, four VRF and a sub-Saharan tourist coming from Equatorial Guinea. All the patients recovered without complications. Data confirmed the higher risk to suffer complicated malaria in children, three out of nine (33.3%), and in pregnant woman, one out of four (25%), but also the increasing of cases of VFR needing admittance in the hospital due to the severity of the symptoms, around 17%. These data suggest that VFR must be included as a risk factor to have complicated malaria in no endemic areas.

The most used treatment pattern was quinine (85.7%) in combination with doxycycline (57.1%) or clindamycin (28.6%). There is no Spanish guideline for malaria treatment and this depends of several factors, including access to the anti-malarial drugs. Most drugs, including quinine, have to be obtained through the Service of Foreign Drugs Supply from the Ministry of Health, as only atavaquone/proguanil and chloroquine are available at chemists. Treatment with artemisinin derivatives is not approved by the Spanish Drug Agency, as is the case in other countries, such as the United States.

It is estimated that only between the 12 to 43% of the travellers to endemic areas follow an anti-malarial prophylaxis, with only 3.1 to 22.8% performing a correct and complete treatment [14,18,26]. For this study, only 10.9% followed some kind of prophylaxis, but only one did correctly with doxycycline. However, this patient acquired a *P. ovale *infection. These data confirms that taking correct prophylaxis is essential to avoid malaria infection.

## Conclusions

Two main conclusions can be drawn from this study. First, that children and pregnant women have the highest risk to present severe malaria and to need hospital admittance, but also the immigrant visiting his/her own country and come back have a high risk to suffer severe malaria. Second, the most important factor responsible for acquiring malaria is incorrect prophylaxis. None of the malaria positive cases took appropriate prophylaxis, except one who acquired *P. ovale *malaria.

A possible solution may be to increase the information given to the sub-Saharan immigrants, which are the highest risk group. This information must include that the semi-immunity acquired by native people noticeably diminishes after living for some months outside an endemic area and, therefore, their risk to acquire malaria increases considerably, and they specially need to take prophylaxis and to follow it correctly.

## Competing interests

The authors declare that they have no competing interests.

## Authors' contributions

SR, IZ and FJM designed the study and compiled data. BMM and JMR reviewed the design on the study. SR and IZ created the databases, ordered and interpreted the results and wrote the article. BMM performed the statistical analysis. JMR performed PCR at CNM. SR, IZ, JMR and FJM performed the bibliographic search and review. FJM and JMR made the final review of the manuscript. All authors read and approved the final manuscript.
